# Dupilumab-Induced Graves’ Disease in an Adult With Chronic Rhinosinusitis and Nasal Polyposis

**DOI:** 10.7759/cureus.89064

**Published:** 2025-07-30

**Authors:** Catarina Gama, Margarida A Oliveira, Bernardo Marques, Pedro Branco, Sequeira Duarte

**Affiliations:** 1 Endocrinology, Diabetes, and Metabolism, Unidade Local de Saúde de Lisboa Ocidental, Hospital de Egas Moniz, Lisbon, PRT; 2 Otolaryngology - Head and Neck Surgery, Unidade Local de Saúde de Lisboa Ocidental, Hospital de Egas Moniz, Lisbon, PRT

**Keywords:** dupilumab, graves’ disease, immune-related adverse effects, monoclonal antibody therapy, th1/th2 imbalance

## Abstract

Dupilumab, a monoclonal antibody targeting interleukin-4 (IL-4) and IL-13, is widely used for the treatment of type 2 inflammatory conditions such as atopic dermatitis, asthma, and chronic rhinosinusitis with nasal polyposis. Although generally well-tolerated, dupilumab has been linked to rare thyroid-related events. We report a case of Graves' disease (GD) in a 47-year-old man with severe nasal polyposis who developed heat intolerance and weight loss within six to eight weeks of starting dupilumab. Laboratory tests confirmed thyrotoxicosis with positive thyroid-stimulating hormone receptor antibodies (TSHR-Ab), leading to a GD diagnosis. The patient was started on methimazole and remains stable on both methimazole and dupilumab. The temporal association between dupilumab initiation and the detection of TSHR-Ab suggests a possible link between dupilumab therapy and the development of GD. This may be mediated by a shift from Type 2 helper T cells (Th2) to Type 1 helper T cells (Th1) immune dominance, which is known to promote autoimmune thyroid activity. Such a mechanism is biologically plausible and consistent with current immunopathogenic models of GD. This case adds to the limited literature on dupilumab-associated autoimmune thyroid disease. Physicians should consider monitoring thyroid function in patients on dupilumab, especially those with immune-mediated conditions. Further studies are needed to clarify this mechanism and guide screening guidelines.

## Introduction

Graves' disease (GD) is the most prevalent cause of hyperthyroidism in iodine-replete geographical areas, with 20-30 annual cases per 100,000 individuals [1]. It is caused by thyroid-stimulating autoantibodies that mimic the action of thyroid-stimulating hormone (TSH) by binding to the TSH receptor (TSHR), leading to hyperthyroidism. GD involves both humoral and cellular immune mechanisms, with contributions from Type 1 helper T cells (Th1), which primarily secrete interferon gamma (IFNγ), and Type 2 helper T cells (Th2), which predominantly produce interleukin-4 (IL-4). IgG1 antibodies arise early in the immune response, whereas IgG4 antibodies (Ab) (typically Th2 related) arise after prolonged immune stimulation. Stimulating TSHR-Ab are mostly found in the IgG1 subclass, which is selectively induced by Th1 cells [2].

Dupilumab, a monoclonal antibody of the IgG4 subclass, is known for its type 2 immunosuppressive properties and is approved for the treatment of atopic dermatitis, asthma, chronic rhinosinusitis with nasal polyposis, eosinophilic esophagitis, and prurigo nodularis [3]. To date, there have been few reports of dupilumab-associated autoimmune endocrine disorders, such as painless thyroiditis accompanied by hypothyroidism [4], type 1 diabetes [5], and hyperthyroidism with negative TSHR-Ab [6]. This case is noteworthy, and this report carries important clinical significance.

## Case presentation

We report a case of a 47-year-old male with a history of severe chronic rhinosinusitis with nasal polyposis. He was resistant to treatment with topical and systemic corticosteroids and antibiotherapy, and relapsed after surgery. He had no other significant medical history other than smoking.

The patient was started on dupilumab’s dose at 600 mg subcutaneously, followed by 300 mg subcutaneously every two weeks. After six to eight weeks of treatment, he began experiencing irritability, weight loss (around 10 kg), and heat intolerance. Laboratory studies revealed a low TSH level (<0.01 mIU/L; normal range: 0.27-4.2) and a high free T4 (FT4) (29.5 pmol/L; normal range: 12.0-24.0). Tests for thyroid peroxidase antibodies (TPO-Ab) and thyroglobulin antibodies (Tg-Ab) were both positive. Subsequently, his family physician began treatment with methimazole at a starting dose of 15 mg/day and referred him to the endocrinology consult.

The patient had no personal or family history of thyroid disease nor a history of iodine supplementation, iodine contrast exams, or amiodarone use. Upon examination, he showed no signs of goiter, neck discomfort, compressive symptoms, or exophthalmos. There were no signs of tremor, agitation, or hyperactivity on physical examination. Vital signs were normal (blood pressure (BP) 135/80 mmHg, heart rate (HR) 84 bpm).

**Figure 1:**
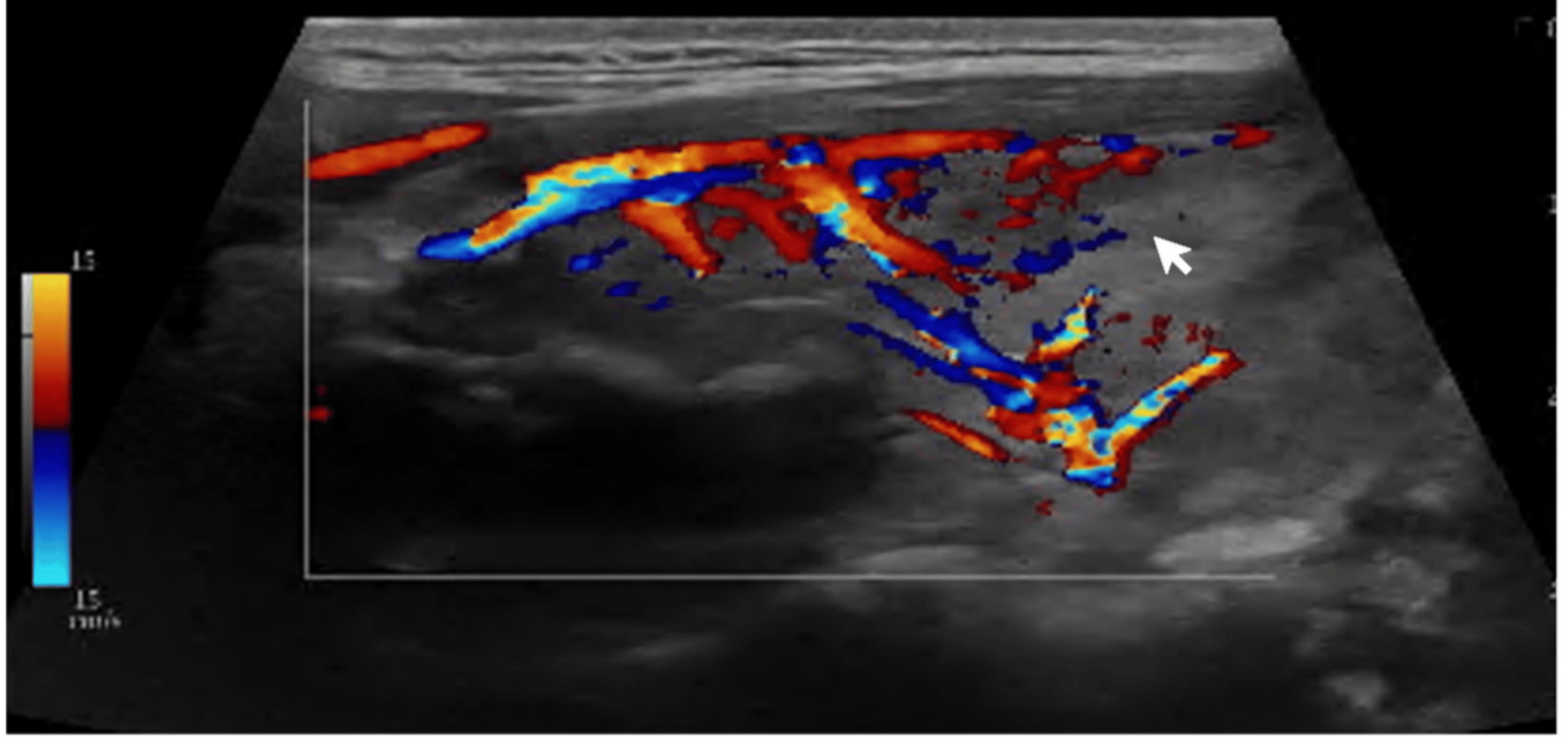
Doppler ultrasound-thyroid (imaging) The Doppler ultrasound image demonstrates an increased vascularity (white arrow).

**Figure 2:**
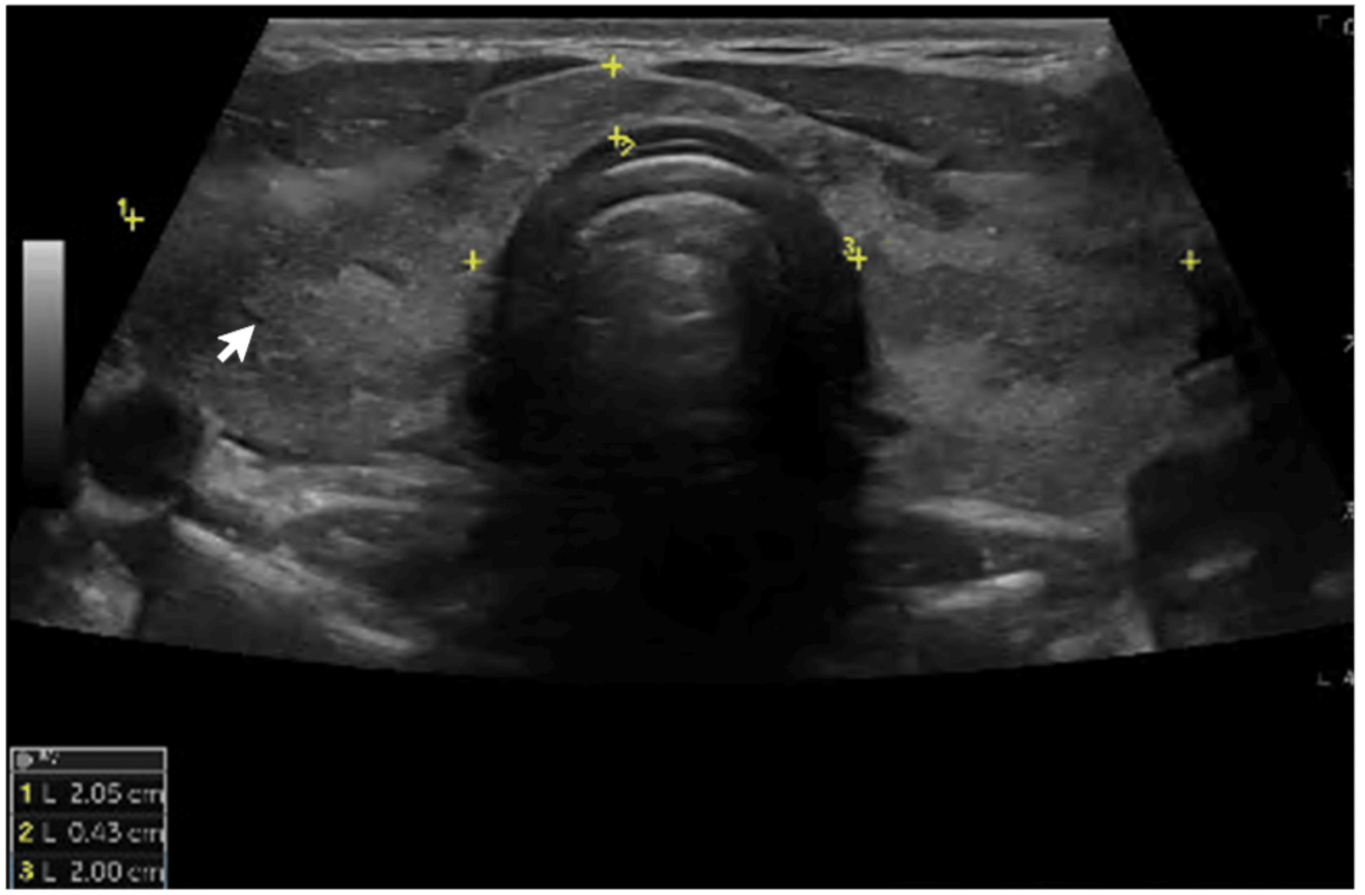
Thyroid ultrasound The ultrasound image demonstrates an enlarged thyroid gland with a diffusely hypoechoic texture (white arrow)

Serum thyroid function tests indicated suppressed TSH (<0.008 mIU/L), with normal FT4 (12.9 pmol/L) and normal free T3 (FT3) (4.92 pg/mL; normal range: 3.10-6.80) levels. Serum measurement of TSHR-Ab confirmed the diagnosis of GD (TSHR-Ab 3.17 U/L; normal range <1.5 U/L). Thyroid ultrasound demonstrated a mildly enlarged gland, with the right lobe measuring 27 × 54 × 22 mm and the left lobe 22 × 53 × 19 mm (transverse, longitudinal, anteroposterior). The parenchyma exhibited mildly heterogeneous echotexture and diffusely increased vascularity, findings that, while non-specific, are suggestive of an underlying autoimmune thyroid process. No thyroid nodules or cervical lymphadenopathy were identified (Figures 1, 2).

The methimazole dosage was initially started at 15 mg/day and then gradually reduced to 5 mg/day. This dosage was maintained for one year, during which the reported symptoms associated with hyperthyroidism gradually improved, leading to progressive weight recovery and resolution of irritability and heat intolerance. The patient was placed under clinical and laboratory surveillance without discontinuation of dupilumab therapy. Dupilumab was continued at a dose of 300 mg every two weeks for one year, with clinical improvement of nasal polyposis. Given this improvement, a trial discontinuation of dupilumab was attempted. Due to clinical deterioration following the discontinuation of dupilumab, the patient has maintained the treatment ever since.

After 12 months of therapy with methimazole, the patient showed no symptoms of hyperthyroidism. Serum measurements indicated TSHR-Ab levels of 0.98 U/L (normal range <1.5 U/L), allowing for the suspension of methimazole therapy. A thyroid ultrasound performed at the time of methimazole discontinuation revealed a thyroid gland of normal dimensions, with a heterogeneous echotexture. No nodules or cervical lymphadenopathy were observed. Doppler evaluation showed reduced vascularity compared to previous examinations.

Six months after stopping methimazole, the patient maintained clinical and biochemical euthyroidism (TSH 0.703 mIU/L, FT4 18.0 pmol/L, FT3 6.04 pg/mL) without treatment. One year after discontinuation of methimazole, the patient remained clinically asymptomatic; however, laboratory testing revealed biochemical relapse with suppressed TSH (0.018 mIU/L), elevated FT4 (18.6 pmol/L) and FT3 (8.46 pmol/L), and reappearance of positive TSH receptor antibodies (TSHR-Ab), indicating disease reactivation. Methimazole was reinitiated at a dose of 5 mg daily, resulting in stable clinical and biochemical euthyroidism.

Given his role as the primary caregiver for his mother, the patient is not currently a candidate for definitive treatment with radioactive iodine (¹³¹I).

Table 1 summarizes the laboratory test results and medication doses over time.

**Table 1:** Summary of laboratory test results and medication doses over time FT3: free T3; FT4: free T4; Tg-Ab: thyroglobulin antibodies; TPO-Ab: thyroid peroxidase antibodies; TSHR-Ab: thyroid stimulating hormone receptor antibodies

Results	Day 0	Week 2	Month 3	Month 9	Month 12	Month 18	Month 24	Normal range
TSH (mIU/L)	<0.01	<0.008	3.80	1.89	3.11	0.703	0.018	0.27-4.2
FT4 (pmol/L)	29.5	12.9	12.0	14.5	17.3	18.0	18.6	12.0-24.0
FT3 (pg/mL)	-	4.92	5.07	5.21	5.51	6.04	8.46	3.10-6.80
TPO-Ab (UI/mL)	>1300	517	-	-	-	-	-	<115
Tg-Ab (UI/mL)	76.0	270.0	-	-	-	-	-	<35.0
TSHR-Ab (U/L)	-	3.17	-	-	0.98	-	1.60	<1.5
Methimazole dose (mg)	-	15	10	5	5	0	0	

After one year of dupilumab therapy, treatment was discontinued; however, a worsening of nasal polyposis occurred one month later, leading to the reintroduction of the medication. Currently, the patient remains on dupilumab therapy, with regular follow-up by the endocrinology team and ongoing otolaryngology follow-up.

## Discussion

The emergence of immunomodulatory therapies has led to remarkable advancements in the treatment of multiple diseases, including cancers and autoimmune disorders. Thyroid dysfunction is a rare immune-related adverse event associated with these therapies; however, the clinical manifestations, severity, and underlying mechanisms can vary significantly. While destructive thyroiditis and hypothyroidism are the most frequently observed immune-related thyroid adverse events induced by immune checkpoint inhibitors, autoimmune hyperthyroidism, such as GD, remains rare [7,8].

Dupilumab is a monoclonal antibody of the IgG4 subclass that inhibits IL-4 and IL-13 signaling by binding to the IL-4 receptor alpha subunit, a component shared by both IL-4 and IL-13 receptor complexes. As a result, the release of pro-inflammatory cytokines, chemokines, and immunoglobulin E is blocked. The most common adverse reactions include injection-site reactions, conjunctivitis, blepharitis, keratitis, eye pruritus, oral herpes, and dry eyes [3]. Few cases of dupilumab-related autoimmune endocrine disorders have been reported. One reported case involved painless thyroiditis accompanied by hypothyroidism [4], while another described dupilumab-associated type 1 diabetes [5], both in patients with atopic dermatitis. Additionally, a pediatric case documented the onset of hyperthyroidism during dupilumab therapy, despite the lack of positive TSHR-Ab [6].

GD has traditionally been viewed as a Th2-driven B-cell disorder. However, evidence indicates that all T-cell types are present in the thyroid glands of patients with GD, suggesting that GD is best understood as a mixture of Th1 and Th2 immune responses [2]. Basil Rapoport et al. propose that early humoral immune response in GD is dominated by IgG1, driven by Th1 cytokines, while prolonged activation is associated with IgG4, promoted by Th2 cytokines. TSHR-Ab, primarily IgG1 and induced by Th1 cells, reinforces the concept of GD as a Th1-associated disorder, challenging the traditional view of it as Th2-dominant [9]. In the active phase of GD, Th1 cells stimulate the production of IFN-gamma and tumor necrosis factor-alpha (TNF-alpha), which induce thyroid follicular cells to secrete CXCL10, driving inflammation. Methimazole treatment shifts this immune response from Th1 to Th2, reducing CXCL10 secretion and the associated inflammation. This suggests that GD is Th1-dominant in its early, active phase and shifts toward Th2 dominance in later, inactive stages [10]. Therefore, dupilumab therapy appears to suppress the Th2 response by inhibiting IL-4 and IL-13 signaling, which may contribute to a relative Th1 dominance, a pattern typically seen in the early immune response of GD.

Our patient was diagnosed with GD after comprehensive testing revealed hyperthyroidism and positive TSHR-Ab, findings that coincided with the initiation of dupilumab therapy. The shift in the Th1/Th2 balance during dupilumab treatment may have contributed to the development of GD in this patient, though this remains speculative. Further cases are needed to help clarify the pathogenic mechanisms linking dupilumab therapy and GD.

Learning points

Dupilumab and Graves' disease: This is an exceptional case report of GD associated with dupilumab treatment, highlighting the need for awareness of potential thyroid-related side effects in patients receiving this therapy.

Th1/Th2 immune shift: GD is typically associated with a Th1-pathway immune response, with Th1 cytokines driving inflammation through TSHR-Ab. Dupilumab's inhibition of IL-4 and IL-13, which are Th2 cytokines, may shift the immune response towards the Th1 pathway, potentially triggering GD in susceptible individuals.

Need for further research: Additional studies are necessary to clarify the mechanism linking dupilumab to GD and to develop screening guidelines for endocrine complications in patients on monoclonal antibody therapies.

Thyroid monitoring recommendations: Given the potential for thyroid dysfunction with dupilumab, clinicians should consider baseline and periodic thyroid function testing in patients on this therapy, especially those with immune-mediated conditions, to enable early detection and management of thyroid-related complications.

## Conclusions

In summary, this is a relevant case report of dupilumab-related GD. The observed amplification of the Th1 pathway, resulting from the inhibition of IL-4 and IL-13, which suppresses Th2 cells, suggests a potential mechanism for GD development. However, the exact mechanisms behind the altered thyroid function remain unclear and require further investigation. Additionally, careful monitoring of thyroid function may also be helpful for individuals undergoing dupilumab treatment. This proactive approach can improve our understanding of the underlying dynamics and enhance patient care by enabling early intervention for potential thyroid-related complications.
